# Reactive Oxygen Species Link Gonadotropin-Releasing Hormone Receptor Signaling Cascades in the Gonadotrope

**DOI:** 10.3389/fendo.2017.00286

**Published:** 2017-10-30

**Authors:** Tomohiro Terasaka, Mary E. Adakama, Song Li, Taeshin Kim, Eri Terasaka, Danmei Li, Mark A. Lawson

**Affiliations:** ^1^Department of Reproductive Medicine, University of California, San Diego, La Jolla, CA, United States; ^2^Neonatal Intensive Care Unit, Dongguan Eighth People’s Hospital Dongguan City, Dongguan, China

**Keywords:** reactive oxygen species, gonadotropin-releasing hormone, pulsatility, DUSP1, gonadotropins, mitogen-activated protein kinase, ERK, pituitary, metabolism

## Abstract

Biological rhythms lie at the center of regulatory schemes that control many aspects of living systems. At the cellular level, meaningful responses to external stimuli depend on propagation and quenching of a signal to maintain vigilance for subsequent stimulation or changes that serve to shape and modulate the response. The hypothalamus–pituitary–gonad endocrine axis that controls reproductive development and function relies on control through rhythmic stimulation. Central to this axis is the pulsatile stimulation of the gonadotropes by hypothalamic neurons through episodic release of the neuropeptide gonadotropin-releasing hormone. Alterations in pulsatile stimulation of the gonadotropes result in differential synthesis and secretion of the gonadotropins LH and FSH and changes in the expression of their respective hormone subunit genes. The requirement to amplify signals arising from activation of the gonadotropin-releasing hormone (GnRH) receptor and to rapidly quench the resultant signal to preserve an adaptive response suggests the need for rapid activation and feedback control operating at the level of intracellular signaling. Emerging data suggest that reactive oxygen species (ROS) can fulfill this role in the GnRH receptor signaling through activation of MAP kinase signaling cascades, control of negative feedback, and participation in the secretory process. Results obtained in gonadotrope cell lines or other cell models indicate that ROS can participate in each of these regulatory cascades. We discuss the potential advantage of reactive oxygen signaling for modulating the gonadotrope response to GnRH stimulation and the potential mechanisms for this action. These observations suggest further targets of study for regulation in the gonadotrope.

## The Challenge of Pulsatile Gonadotropin-Releasing Hormone (GnRH) Signaling in Gonadotropes

The fundamental role of pulsatile stimulation of gonadotropes by the hypothalamic neuropeptide GnRH, or GnRH-I in maintaining function of the hypothalamic–pituitary–gonad (HPG) axis is one of the earliest principal findings after discovery of the hormone (1, 2). Studies in nonhuman primates demonstrated the requirement for pulsatile stimulation of the pituitary to maintain the reproductive axis (3). The identity of the signaling molecules and mechanisms that contribute to pulse interpretation has been the subject of extensive study since many models developed to explain signaling control of gene expression (4–13). But questions remain concerning the mechanism of pulse interpretation and the signaling factors responsible. The hypothalamic neuropeptide GnRH and its receptor GnRHR are the prototypic members of a superfamily that has evolutionary roots reaching to the emergence of the bilateria (14). In vertebrates, a feature of this pair is its central role in regulating the anterior pituitary gonadotropes (15, 16). The hypothalamic GnRH neurons release hormone into the adenohypophyseal portal circulation in an episodic manner that is central to the development and operation of the HPG axis and fertility. In this system, the GnRHR governs the release of the gonadotropins LH and FSH and regulates expression of their subunit genes. The mammalian GnRHR is unique in structure, lacking a cytoplasmic tail that is normally associated with β-arrestin-mediated downregulation of receptor signaling. Thus, GnRHR itself faces unique challenges in transmitting an episodic signal in which alterations in amplitude and frequency are meaningful, yet, receptor homologous desensitization is not an accessible regulatory scheme. It is likely that pulse interpretation is accomplished by the operation of the signaling cascades themselves rather than desensitization or receptor availability at the membrane.

In mouse LβT2 cells, the switch between LH and FSH preference occurs at the 60-min pulse interval (17). A general switching mechanism is achieved by the expression and decay of activating and repressing transcription factors that create high- or low-pass filters to govern gene expression. For *Lhb*, this is the pairing of the immediate-early *Egr1* family of transcriptional activators with the Nab1/2 family of repressors. Transient frequency and amplitude-dependent stimulation of *Egr1* expression is countered by pulse-insensitive expression of *Nab*1/2, establishing a high-pass filter that requires sustained stimulation to overcome suppression (17). Features of this model have been confirmed by *in vivo* studies and mixed primary pituitary culture in rats and in αT3-1 cells that do not express gonadotropin β-subunit genes (18). On the other hand, *Fshb* prefers low frequencies for promoter activator (*c-Fos and c-Jun*) upregulation, and high frequencies lead to upregulation of *Fshb* promoter inhibitors, such as *Skil* and *Tgif1* (19). A feature of pulse decoding in the GnRH system is the occurrence of maximal responses at submaximal stimulation, creating a bell-curve frequency response that requires complex regulation but imparts true frequency decoding (20, 21). Components of the signaling network may exhibit digital tracking in which each response is resolved between pulses and acts dependently, or in the case of slower, incomplete resolution, exhibits integrative tracking in which the cumulative stimulation creates a maximal response (10). Transcriptional regulation of gonadotropin subunit genes is modest overall but exhibits integrative interpretation (22). GnRH also regulates protein synthesis and the distribution of mRNA in polyribosomes (23–26). Each of these may utilize different interpretative mechanisms.

## MAP Kinase Signaling in Response to GnRH

**Figure 1:**
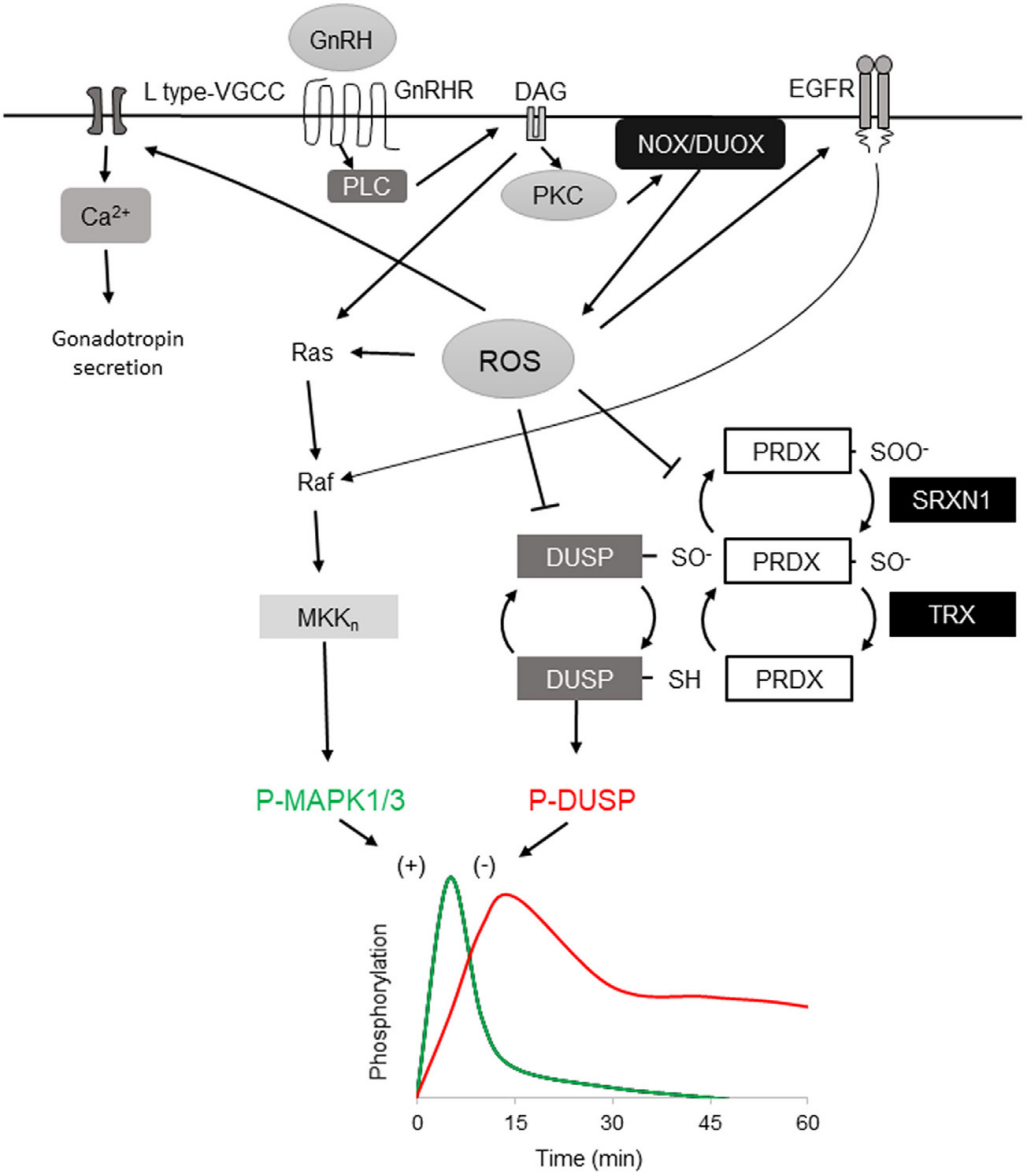
Reactive oxygen species (ROS) involvement in mitogen-activated protein kinases (MAPK) 1/3 activation by gonadotropin-releasing hormone (GnRH) and resolution in gonadotrope cells. Activation profiles of MAPK1/3 and DUSP1 as determined by phosphorylation in response to a single GnRH pulse [adapted from Ref. (35)]. GnRH receptor-signaling *via* G_aq/11_ activates phospholipase C, leading to diacylglycerol (DAG) and IP_3_ production. The DAG and IP_3_-induced rise in intracellular Ca^2+^ activate both NOX and DUOX family members, resulting in increased ROS production. ROS stimulates MAPK1/3 activation by promoting Ras and Raf activation of the MEKn cascade ultimately targeting MAPK1/3. Oxidative activation of epidermal growth factor receptor (EGFR) contributes to MAPK1/3 activation through Raf. ROS may also transiently inactivate negative feedback through reversible oxidation of the DUSP active-site cysteine. ROS is normally reduced by peroxiredoxin (PRDX) by conversion of active reducing site cysteine thiol C–SH to sulfenic C–SOH. Sulfenic cysteine is recycled by thioredoxin (TRX) reduction. Excess ROS contributes to PRDX hyperoxidation that further oxidizes the sulfenic C–SOH to the sulfinic C–SOOH, which is reduced by the ATP-dependent reductase activity of sulfiredoxin 1 (SRXN1), preserving PRDX capacity but allowing transient DUSP inactivation. DUSP activity is resumed after ROS level declines, permitting feedback control of MAPK1/3. ROS activation of L-type VGCC promotes intracellular Ca^2+^ that supports exocytosis and activation of DUOX.

GnRHR is a G protein-coupled receptor that signals primarily *via* the G_αq/11_ G protein subfamily, although interaction with other G proteins is also documented *in vivo* (27, 28). Stimulation of gonadotropes or gonadotrope-derived cell lines causes activation of phospholipase C, resulting in inositol 1,4,5-trisphosphate (IP_3_) and diacylglycerol (DAG) production. IP_3_ mobilizes Ca^2+^ from intracellular stores and influx *via* L-type voltage-gated Ca^2+^ channels. The mobilization of Ca^2+^ is associated with initiation of the secretory response and fusion of secretory granules with the extracellular membrane. In a related signaling branch, DAG along with Ca^2+^ activates multiple PKC isozymes, including the conventional isoforms PKCα, PKCβII, the novel isoforms PKCδ and PKCε, and the atypical PKCζ in αT3-1 and LβT2 cells (29, 30). These activated signals link to downstream induction of mitogen-activated protein kinases (MAPK) (18, 31–33). The role of MAPK1/3 (ERK1/2) is sexually dimorphic and essential in female reproduction (34). Phosphorylation of MAPK1/3 is highly stimulated within a few minutes and rapidly resolved such that MAPK1/3 activation is restored to prestimulation levels well within the 60-min interval switch point of differential gene expression (Figure 1) (35). The connection between PKC and MAPK1/3 activation is well appreciated, but the intervening sequence of Ras/Raf/MAPK kinase (MEKK) signaling is not well described (29, 30). MAPK1/3 activation can occur through the c-SRC-mediated RAS activation (30, 36) and, in other cells, RAS activation occurs through DAG-dependent GRP1/2. However, recent evidence has shown that GnRH-stimulated MAPK1/3 activation in gonadotropes depends on reactive oxygen species (ROS) production by the NADPH oxidases (37). This suggests that multiple pathways contribute to MAPK1/3 activation and examination may shed light on their contribution to pulse interpretation.

## ROS Integration into Pathways Promoting MAP Kinase Signaling

Reactive oxygen species are partially reduced metabolites of oxygen produced through intracellular mechanisms or encountered in extracellular environments. Mitochondrial ROS are produced by aerobic respiration and incomplete oxidation of fatty acids and can indicate mitochondrial and endoplasmic reticulum stress. ROS are also employed as rapid signaling molecules through production by the NADPH/Dual Oxidase (NOX/DUOX) family, which are targets of activation by intracellular kinases or elevated intracellular Ca^2+^ (38). Exposure to ROS can cause oxidative damage to many biomolecules, resulting in nucleic acid damage or mutation, enzymatic dysfunction, or cell death. Therefore, management of ROS is a focus of cellular homeostasis. Reducing systems operating through peroxiredoxins (PRDX1-6), thioredoxin (TRX), and glutathione (GSH) exchange of free radical oxygen are present in all cells and PRDX isoforms partition into subcellular regions for specialized action. Each of the six mammalian PRDX isoforms is represented in to the top 5% of cellular protein content (39), collectively constituting a high proportion of cellular protein and a significant investment in localizing ROS action and limiting oxidative damage.

Elevated ROS is associated with MAPK activation in multiple cell types (40). Insulin-like growth factor I activation of MAPK1/3 increases ROS production and antioxidants inhibit activation of the MAPK1/3 pathway, showing dependence on ROS (38–41). Similarly, MAPK8/9 (JNK) and MAPK14 (p38 MAPK) phosphorylation is associated with ROS generation (42–44). The MAP3K-related kinase ASK1 associates with TRX and is released upon TRX oxidation, permitting activation of MAPK8/9/14 (44). In other professional secretory cells, ROS is central to secretion and activation of biosynthesis. In the endocrine pancreas, NOX enzymes are involved in stimulated insulin secretion and excess ROS production increases oxidative stress and loss of function (45–47). NOX/DUOX participate in the signaling response activating thyroid hormone biosynthesis (48–50). ROS mediates enhanced MAP kinase activation in activated eosinophils, contributing to IL-5-mediated cell death (51). In contrast, NOX is a target of MAP kinase activation in neutrophils and ROS signaling is utilized in formation of neutrophil extracellular traps (52). NOX proteins are, therefore, both upstream activators of MAP kinase signaling and targets of MAP kinase action, suggesting plasticity in how NOX/DUOX-derived ROS is deployed.

Gonadotropin-releasing hormone stimulation of mouse primary pituitary and LβT2 cells that endogenously express all gonadotropin subunit genes (53, 54), results in ROS production that is blocked by pharmacological inhibition of NOX/DUOX enzymes with diphenyleneiodonium (DPI). N-acetyl cysteine (NAC), which is general ROS scavenger, also attenuates MAPK1/3 and MAPK8/9 activation. Both DPI and NAC attenuate activation of *Lhb* and *Fshb* transcription (37). Further, GnRH-mediated activation of ROS depends on PKC and Ca^2+^ availability. This places ROS between PKC and MAPK1/3 and supports an intermediate role in activation similar to ROS-mediated activation of Ras through kinase and regulatory subunit regulation (55, 56). The rapid and transient activation of MAPK1/3 by GnRH is similar to that reported by direct activation by H_2_O_2_ *via* epidermal growth factor receptor (EGFR) (57). The cysteine-rich motifs of growth factor receptors including EGFR are proposed targets of activation by oxidation (42). But demonstration using suramin and its known broad actions that include inhibition of G-protein receptor signaling suggests that revisiting this may be warranted. GnRH signaling is also associated with EGFR activation possibly through matrix metalloprotease liberation of extracellular ligand (58–60). An alternative ligand-independent activation pathway through oxidative activation of EGFR could support the rapid elevation of MAPK 1/3 activation after GnRH stimulation (41).

In addition to activation of MAP kinase pathways through positive regulation of signaling cascades, ROS may also play a central role in promoting MAP kinase phospho-activation through inactivation of negative feedback. Dual-specificity protein phosphatases (DUSP’s, also MKP’s) serve a primary role as negative feedback regulators of MAP kinase signaling through dephosphorylation of activated MAP kinases (Figure 1) (61). DUSP’s and other protein tyrosine phosphatase superfamily members share a common catalytic site motif of [I/V]HCXXGXXR[S/T] in which the invariant cysteine residue serves as a catalytic nucleophile that is susceptible to reversible inactivating oxidation (62). Oxidative suppression of DUSP’s can support sustained activation of MAPK 8/9 and drive TNF-α-mediated cell death (63) and oxidative control of DUSP and MAPK signaling has been observed in pancreatic β-cells (46, 64), supporting this mechanism in professional secretory cells. Oxidation of DUSP’s also promotes their proteosomal degradation, limiting their availability to inhibit MAPK kinase (65). In LβT2 gonadotropes, high amplitude GnRH stimulation causes sustained activation of MAPK1/3 similar to that observed with ROS-mediated suppression of DUSP feedback (35). Chronic stimulation with GnRH also results in ROS production (37) but the status of DUSP after prolonged exposure to ROS or chronic GnRH stimulation has not been directly examined. The participation of ROS in rapid activation of MAP kinase signaling in gonadotropes through positive control of signaling cascades and negative control of feedback suggests that ROS contributes to the rapid activation of MAP kinases that is observed in response to GnRH stimulation. Involvement in both MAPK 1/3 and MAPK 8/9 activation suggests that both *Fshb* and *Lhb* transcription can be regulated through ROS.

## Resolution of GnRH-Stimulated MAPK Signaling

Feedback control of MAPK activation by DUSP family members is central to the control of MAP kinase signaling networks. For cells to remain vigilant for change in GnRH pulses, sensitivity to a subsequent pulse is maintained and interpreted in context, which implies a capacity for hysteresis. In either digital or integrative pulse tracking, some balance between activation, negative feedback, and response decay must be achieved. Signaling networks may switch between modes by changing this relationship. Hysteresis in cell signaling was initially proposed and tested in the model of bistable MAPK1/3 activation by platelet-derived growth factor receptor, which showed that MAPK1/3 response amplitude is dictated by the degree of DUSP1 feedback activated by a previous signaling response (66). The role of DUSP’s in negative feedback control of MAPK1/3 activation has been examined extensively in the context of GnRHR signaling. In LβT2 cells, activation of MAPK1/3 by physiological levels of GnRH is resolved within 30 min (35). Overexpression or knockdown of nuclear-resident DUSP1 suppresses or increases activation of MAPK1/3 in response to GnRH, respectively (35). But studies in cells that do not natively express GnRHR or gonadotropin genes or using reporters of translocation have questioned this observation (67). LβT2 gonadotropes show elevated DUSP1 in unstimulated cells, suggesting that they are primed for suppression of MAP kinase signaling activation. This available phosphatase activity is subject to rapid inactivation by ROS but the reversibility of inactivation suggests some capacity is maintained or quickly recovered, contributing to the rapid resolution of MAPK1/3 activation. Another possibility is the involvement of cellular mechanisms limiting ROS through reduction by PRDX and TRX. These proteins are part of a larger network of factors controlling oxidative stress that includes GSH, catalase, superoxide dismutase, and the ATP-dependent redox factor sulfiredoxin 1 (SRXN1, also NPN3). These factors contribute to the maintenance of reductive capacity through resolution of oxidized or hyperoxidized PRDX, returning it to the pool of available reductase. Although the 2-cysteine PRDX1-4 family members are efficient ROS scavengers, they can be hyperoxidized by conversion of their nucleophilic thiol to sulfinic acid (Figure 1). Hyperoxidized PRDX is recycled through ATP-dependent reduction by SRXN1 (68, 69). In LβT2 cells, *Srxn1* gene expression is proportionally induced by increasing pulsatile and tonic GnRH stimulation (17), implying a role in resolution of oxidative stress.

The restoration of feedback control can also be achieved through increased DUSP synthesis in response to GnRH stimulation. In LβT2 cells, GnRH stimulation causes transient activation of the unfolded protein response (UPR) (24). Translation is largely inhibited by the UPR but *Dusp1* and *Dusp8* mRNA escape translation inhibition and DUSP1 is increased during the time the UPR is active (23, 35). Translational control of *Dusp1* mRNA is MAPK1/3 dependent and is attributed to the 3′UTR ELAVL1 binding site known to contribute to mRNA stability (23, 70). Pulsatile GnRH increases *Dusp1, Dusp8*, and *Dusp16* expression, all of which target MAP kinases (17). Although DUSPs are subject to rapid inactivation by ROS, the reversibility of oxidative inhibition, the rapid translational response of the UPR, and the long-term transcriptional response to GnRH stimulation provide mechanisms for preserving feedback regulation while permitting short-term activation of MAP kinase signaling.

On a broad time scale, ROS may regulate gonadotropins through regulation of gene expression and sensitivity to GnRH through microRNA modulation of gonadotropin and G_αq/11_ signaling component gene expression. MiR132/212 regulates *Fshb* mRNA expression and secretion *via* SIRT1 deacetylation in gonadotropes (71). MiR-7a2 or miR-200b and miR-429 participate in maintenance of gonadotropin gene expression (72, 73). Further, miR125b contributes to desensitization of sustained GnRH stimulation by targeting components of the G_αq/11_ pathway (74). MicroRNA regulation occurs through oxidation-sensitive transcription factors such as CEBPB and ZEB1 and microRNA also directly affects MAPK signaling (75). The ROS-sensitive regulation of microRNA may further link GnRH signaling to ROS directly through GnRH receptor stimulation of ROS production or to ROS derived from other sources.

## ROS in GnRH-Induced Gonadotropin Secretion

Hormone secretion by exocytosis in endocrine cells is triggered by Ca^2+^ released from intracellular stores. A rise of intracellular Ca^2+^ regulates several steps of exocytosis; including, vesicle priming and fusion to the plasma membrane (76). Localized Ca^2+^ increase with IP_3_ stimulation is necessary for gonadotropin exocytosis (77). Increased Ca^2+^ also induces ROS production by DUOX activation (37) and both voltage-gated and L-type calcium channels are activated by ROS (78, 79). This may tie localized Ca^2+^ to enzymatic ROS generation by DUOX. Thrombin promotes Ca^2+^ influx in smooth muscle cells by NOX-derived ROS activation of L-type calcium channels (80). Also, insulin-induced NOX increases IP_3_ receptor activity and Ca^2+^ release in skeletal muscle (81). In gonadotrope cells, DPI blocks GnRH-induced gonadotropin secretion, indicating dependence on ROS for secretion (37) and suggesting integration of Ca^2+^ and ROS signaling in exocytosis.

Interestingly, ROS in the form of nitric oxide may play an important role in maturation and regulation of the hypothalamus in concert with pituitary ROS. Nitric oxide production in the hypothalamus elicits GnRH secretion and expression of GnRH mRNA is modulated through miR-200 and miR-155 expression before puberty by controlling the nitric oxide-sensitive regulators CEBPB and ZEB1 (82, 83). These phenomena indicate that microRNA and ROS are tightly linked to rapid and long-term control of the HPG axis.

## ROS as a Metabolic Reporter in the Gonadotrope

**Figure 2:**
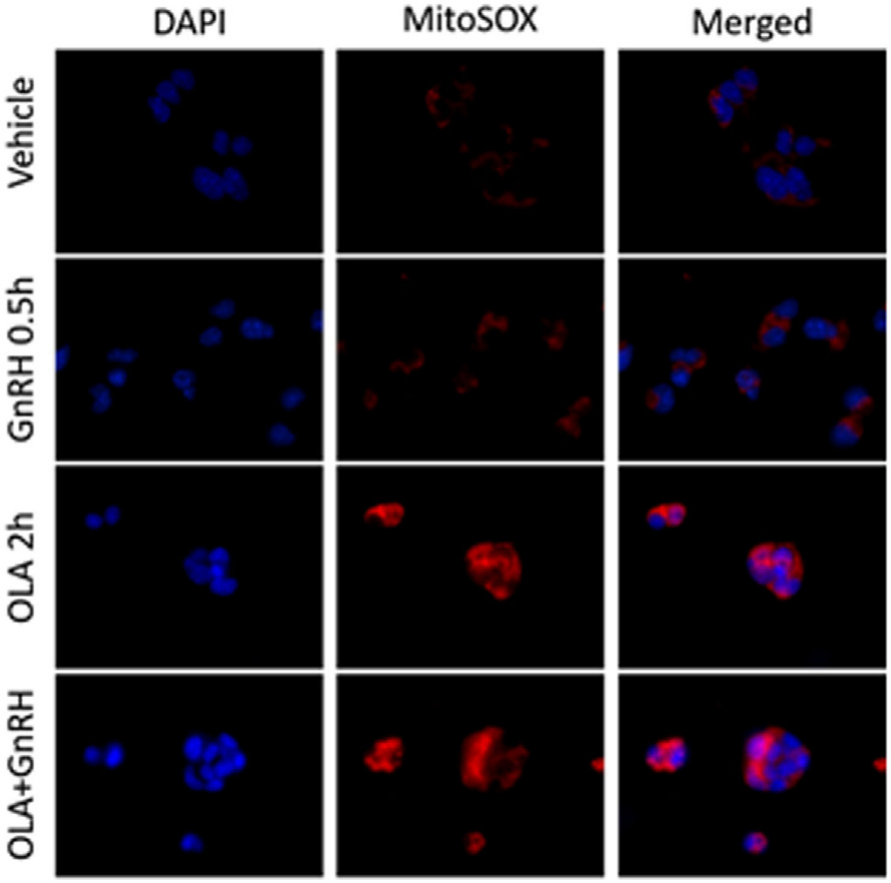
Oleate (OLA), but not gonadotropin-releasing hormone (GnRH), induces mitochondrial superoxide production. LβT2 cells (RRID:CVCL_0398) cultured on Poly-l-Lysine (Sigma-Aldrich Inc.) coated Nunc Lab-Tek II chamber slides (Thermo Fisher Scientific) for 24 h were serum starved overnight, then exposed to either vehicle or 500 µM oleate for 2 h, then with vehicle or 10 nM GnRH for 30 min. Cells were subsequently treated with 5 µM red-fluorescent MitoSOX probe (Thermo Fisher Scientific) for 5 min. Afterward, cells were directly fixed with 2% paraformaldehyde solution for 15 min, washed, and cover slipped with mounting medium containing 4′,6-diamidino-2-phenylindole (DAPI) (Vector Labs) to visualize nuclei in blue. Blue DAPI and Red MitoSOX fluorescence was captured by wide-field fluorescent microscopy using Nikon TE2000-U microscope (Nikon America Inc., Melville, NY, USA) equipped with an X-Cite 120PC collimated light source (Lumen Dynamics Group Inc.) and a DAPI-1160A or mCherry-C000 filter set (Semrock, Inc.) using a CoolSNAP DYNO CCD camera (Photometrics Inc.). In LβT2 cells, GnRH alone does not enhance mitochondrial ROS production as determined by changes in red fluorescence, whereas OLA-treated cells showed a highly elevated signal. Co-treatment with GnRH and Oleic acid did not appear to alter overall staining intensity.

Energy balance has a profound influence on reproductive fitness and operation of the HPG axis. The critical fat hypothesis suggests that an optimal level of body fat is permissive to menarche and may be necessary for optimal operation of the HPG axis (84, 85). Adipose-associated changes in gonadotropin levels imply the presence of a sensing mechanism that reports energy status to the reproductive endocrine axis (86–91). Reproductive disorders such as polycystic ovary syndrome and hypogonadotropic hypogonadism are associated with metabolic dysfunction and obesity. However, not all metabolic signals associated with obesity explain the inverse relationship between adiposity and gonadotropin levels observed in men and women (92–95). Adipose-derived endocrine signals such as leptin play a role in modulating hypothalamic or pituitary function (96). Another potential modulator of the HPG axis are free fatty acids (FFA). Data suggest that FFA have a direct impact on gonadotropes in ruminants, and FFA suppresses gonadotropin secretion in cultured primary pituitary (97, 98). Unsaturated FFA can induce mitochondrial ROS production and activation of the UPR (99). We examined the ability of the monounsaturated fatty acid oleate (OLA) to induce mitochondrial ROS in LβT2 cells (Figure 2). We found that moderate physiological OLA, 500 µM, can induce mitochondrial. Unlike OLA, GnRH does not impact mitochondrial ROS production as measured by conversion of the indicator dye MitoSOX nor does there appear to be any synergistic action, although GnRH induces ROS production in the same cells *via* NOX/DUOX (37). This supports the enzymatic ROS production with GnRH stimulation, and casts us the question that mitochondria produced ROS needs to be further examined in the aspect of both gonadotropin secretion and GnRH-induced gene expressions.

## Conclusion

The emerging evidence that ROS are integrated into multiple cell signaling cascades has led to appreciation as an important signaling molecule. In gonadotropes, it appears that enzymatic ROS plays a role in GnRH receptor signaling. The rapid and transient nature of ROS signaling is well-suited for the episodic pattern of GnRH stimulation and ROS signaling can contribute to both rapid activation and rapid resolution of activated signaling cascades. Further studies are needed to confirm or disprove the role of PRDX and SRXN1 in resolution of MAPK activation. It is also necessary to examine the impact of FFA-induced ROS on cell stress and to confirm the potential role of enzymatic ROS on L-type calcium stimulation in gonadotrope cells. The integration of ROS in GnRH receptor signaling provides an opportunity for other ROS sources to impact gonadotrope function at the cellular level. Incorporating ROS into our view of GnRH signaling is likely to yield useful insight into the mechanism of pulse interpretation and integration of stress signaling into the reproductive axis.

## Author Contributions

TT authored the manuscript and prepared figures. MA produced new data presented in Figure 2 under direction and technical support of TT and DL. TK and SL developed the original concept, experimental approaches, and produced pilot data not reported here. ET participated in editing and writing the manuscript. ML edited the manuscript and secured support for all co-authors.

## Conflict of Interest Statement

The authors declare that the research was conducted in the absence of any commercial or financial relationships that could be construed as a potential conflict of interest.

## Abbreviations

DAG, diacylglycerol; DPI, diphenyleneiodonium; DUOX, dual oxidase; DUSP, dual-specificity protein phosphatase; EGFR, epidermal growth factor receptor; FFA, free fatty acids; GnRH, gonadotropin-releasing hormone; GnRHR, gonadotropin-releasing hormone receptor; GSH, glutathione; HPG, hypothalamic–pituitary–gonad; IP_3_, inositol 1,4,5-trisphosphate; MAPK, mitogen-activated protein kinase; MEKK, MAPK kinase; NAC, N-acetyl cysteine; NOX, NADPH oxidase; OLA, oleate; PRDX, peroxiredoxin; ROS, reactive oxygen species; SRXN1, sulfiredoxin 1; TRX, thioredoxin; UPR, unfolded protein response.
